# Editorial: Sudden Unexpected Death in Epilepsy: Bio-markers, Mechanisms, Risk Identification and Prevention

**DOI:** 10.3389/fneur.2019.01277

**Published:** 2019-12-04

**Authors:** Rainer Surges, Michael R. Sperling, Christopher M. DeGiorgio

**Affiliations:** ^1^Department of Epileptology, University Hospital Bonn, Bonn, Germany; ^2^Department of Neurology, Jefferson Comprehensive Epilepsy Center, Thomas Jefferson University, Philadelphia, PA, United States; ^3^Department of Neurology, David Geffen-UCLA School of Medicine, Sylmar, CA, United States

**Keywords:** sudden death, blood pressure, apnea, sleep, ketogenic diet, epilepsy, seizures, mortality

Sudden unexpected death in epilepsy (SUDEP) is a common cause of epilepsy-related mortality, with an incidence of 1.2 per 1,000 persons (1). SUDEP causes 15–17% of all epilepsy-related deaths and carries a lifetime risk of 4–8% (1, 2). Reliable biomarkers and predictors are still lacking, but the most important risk factors are nocturnal and frequent tonic-clonic seizures. Mechanisms include tachycardia and tachypnea followed by bradycardia, asystole, and apnea in SUDEP cases documented in epilepsy monitoring units (3). The underlying neuronal and molecular pathomechanisms that turn non-fatal into fatal seizures remain to be elucidated, but brainstem dysfunction and spreading depression as well as defects in serotonin-related signaling appear to be involved. Comprehensive information about SUDEP is recommended and desired by most patients, relatives, and caregivers. Improved seizure control by pharmacotherapy, epilepsy surgery, and neuromodulatory devices is key to SUDEP prevention. Nocturnal supervision is associated with reduced SUDEP risk, and clinically tested seizure detection systems are available.

## Cardiorespiratory Mechanisms

Postictal breathing disturbances are a hallmark of monitored SUDEP cases (3). In this Research Topic, Vilella et al. investigated the occurrence and influencing factors of seizure-related central apnea in a prospective multicenter study. They found that postictal central apnea occurred in about 20% of tonic-clonic seizures (generalized convulsive seizures) in 20% of the patients, its recurrence risk amounted to about 50% in a given individual. Postictal central apnea was less frequent than ictal central apnea and seen in all epilepsy types, possibly indicating different pathomechanisms. In addition to respiratory dysfunction, recent studies described profound alterations of systemic arterial blood pressure and its regulation in association with seizures [e.g., (4, 5)], suggesting a possible role in SUDEP (6). Nass et al. summarize the current knowledge on control of blood pressure in epilepsy, describe the relevant cerebral mechanisms and pathways and discuss how epilepsy- and seizure-related blood pressure alterations may contribute to premature mortality and SUDEP.

## Risk Factor “Nocturnal Seizures” and Nutrition

Seizures arising from sleep or occurring during nighttime were identified as a risk factor for SUDEP (7). To date, it is still unresolved whether this is predominantly due to the fact that people are more often unsupervised or to distinct neuronal mechanisms and networks properties related to sleep. Purnell et al. review the possible factors contributing to the relationship between sleep and SUDEP and explain potential molecular and neuronal mechanisms. In a mouse model of Dravet syndrome with high mortality, Teran et al. observed that SUDEP rates and seizure frequency were higher in the early evening and nighttime, suggesting that specific circadian or sleep-related neuronal mechanisms increase the risk of SUDEP. They also noted that placing mice on a ketogenic diet led to a considerable reduction in mortality. Importantly, the mice fed with ketogenic diet did not have fewer seizures than control mice, indicating that nutritional effects on mortality and the SUDEP rate were independent of seizure frequency. In another experimental study of this Research Topic, Taha et al. tested the effects of dietary measures on seizure thresholds in rat models of kindling and acute seizures. They found that chronic dietary omega-3 polyunsaturated fatty acid deficiency did not alter seizure thresholds, but impaired the effects of acutely applied omega-3 docosahexaenoic acid (Taha et al.) Altogether, the results of these two experimental studies confirm that nutrition has an impact on brain excitability and underscore that more research should be performed on diet in the context of epilepsy and maybe SUDEP.

## Biomarkers, Risk Identification, and Prevention

The search for reliable biomarkers and predictors of an elevated SUDEP risk is of great importance for designing appropriate safety measures and interventional studies to prevent SUDEP. An increasing number of studies deals with the question whether brain regions involved in the regulation of autonomic function display functional and structural alterations that are linked to the SUDEP risk. If present, these alterations could serve as neuroimaging markers that would help to identify people at higher risk. In this Research Topic, Allen et al. have compiled recent findings on resting-state functional and structural MRI studies, strengthening the view that disturbed central autonomic and respiratory control contributes to the pathophysiology of SUDEP. For instance, studies on morphometry and cortical thickness revealed reduced volume and cortical thinning in the thalamus, frontal cortex and at brainstem sites in patients with SUDEP and in those with tonic-clonic seizures. Importantly, the authors point out that the MRI-based prediction of the individual SUDEP risk requires further studies. A personalized estimation of the danger, however, would be very helpful when counseling people with epilepsy and their relatives or caregivers. In this context, Shankar et al. ask if “the time has come to stratify and score SUDEP risk to inform people with epilepsy of their changes in safety”. The authors present work which identifies the need to improve communication at a primary care level. They suggest that regular reviews using a structured risk factor checklist as a screening tool would help in earlier identification of people whose health is worsening and to justify referrals to specialists.

## Summary

This Research Topic compiles recent findings on seizure-related breathing disturbances and blood pressure regulation, reviews the impact of sleep and nutrition on brain excitability and SUDEP and summarizes our current knowledge on neuroimaging markers. The variety of the contributions illustrates that an increasing number of clinicians and researchers are committed to understand SUDEP, identify biomarkers and develop strategies and interventions to mitigate the SUDEP risk. These worldwide efforts are also the success of tireless activities of patients, families and bereaved to promote awareness for premature mortality in epilepsy and SUDEP (8).

## Author Contributions

RS drafted the editorial. MS and CD critically revised the draft.

### Conflict of Interest

The authors declare that the research was conducted in the absence of any commercial or financial relationships that could be construed as a potential conflict of interest.
